# A scoping review of adult NCD-relevant phenotypes measured in today’s large child cohort studies

**DOI:** 10.1038/s41390-025-04056-3

**Published:** 2025-04-12

**Authors:** Katie McBain, Dorothea Dumuid, Ashleigh Shipton, Susan A. Clifford, Timothy Olds, Melissa Wake

**Affiliations:** 1 Murdoch Children’s Research Institute, Royal Children’s Hospital, Parkville, VIC Australia; 2 Department of Paediatrics, The University of Melbourne, Parkville, VIC Australia; 3 Alliance for Research in Exercise, Nutrition and Activity, Allied Health and Human Performance, University of South Australia, Adelaide, SA Australia

## Abstract

**Background:**

Child cohort studies are important resources that can inform strategies to prevent adult noncommunicable diseases (NCDs). Technological advances now enable direct measurement of NCD-relevant phenotypes at large scale. Across contemporary large child cohorts, we aimed to provide the first comprehensive map of NCD-relevant phenotype measurement and gaps.

**Methods:**

We included cohorts with >8000 child participants that were recruiting in whole or part after 2010 and measuring phenotypes relevant to ten high-burden NCDs. Our database and gray literature search identified 15 cohort studies for inclusion. Details on phenotype measurement (methods, age, location) are presented in an online, searchable inventory.

**Results:**

All 15 cohorts measure body size or composition. Most cohorts measure aspects of cardiovascular health (*n* = 10) and neurocognition (*n* = 9). Fewer measure musculoskeletal phenotypes (*n* = 6), pulmonary function (*n* = 6), vision (*n* = 6) and glucose (*n* = 4). Only two cohorts measure hearing or kidney function.

**Conclusions:**

Today’s childhood cohorts are not measuring some phenotypes important to global burden of disease, notably kidney function and hearing. Given the rarity of very large contemporary child cohorts, cross-cohort coordination will be required if all major NCD precursors are to be adequately represented for future benefit.

**Impact:**

- This scoping review provides a comprehensive overview of NCD-relevant phenotype measurement across large, modern child cohort studies.
- This review has identified measurement gaps in important areas that may obviate steps to prevent and detect NCDs with high global disease burden.
- Findings may inform planning of collaborative projects and future data collection to address measurement gaps for greatest future benefit.

## Introduction

Noncommunicable diseases (NCDs) are the leading cause of death worldwide, accounting for more than 70% of all deaths, and contribute enormously to global disease burden.^1,2^ In Australia, as in other high-income countries, approximately 90% of deaths are caused by NCDs.^3,4^ Ten conditions of aging account for 59% of global burden of disease in adults aged 75 and older: cardiovascular diseases (ischaemic heart disease, stroke and hypertensive heart disease, 31.6%), chronic obstructive pulmonary disease (COPD, 8.5%), Alzheimer’s disease (5.6%), diabetes mellitus (4.0%), fractures resulting from falls (2.6%), chronic kidney disease (2.5%), age-related hearing loss (2.5%) and blindness and vision loss (1.7%).^2^ Since 2010, gains in life expectancy in high-income countries have either plateaued or fallen, largely due to the rise of NCDs and their risk factors.^5,6^ Action is needed for prevention and early detection of NCDs but, to do this, we need to understand the life course trajectories to these conditions.

Cohort studies are valuable resources that, through measurements over time, capture the exposures, antecedents and outcomes of NCDs. Cohort studies that recruit participants during pregnancy, childhood or adolescence and track participants as they develop are well placed for discoveries that could elucidate life course phenotype trajectories that underlie adult NCDs.^7^ All cohort studies must make choices as to their areas of focus. As no one cohort can measure everything, it is important that collectively these resources span critical areas relating to current and future health. Concurrent burden of childhood disease/disability, wellness and salutogenesis are all vital research areas; another is measuring early life phenotypic pathways to later life disease – the focus of this paper. Phenotype trajectories reflect the functioning of various body systems (e.g., pulmonary function, neurocognitive function) across the life course at the organ level.^8^ They provide insights into the pathophysiological changes preceding later disease and highlight the role of early exposures and risk factors. Trajectories offer opportunities to study mechanisms for their positive disruption and to target interventions during optimal windows of growth, peak and decline.^9^

Many cohort studies now combine self- or proxy-reported measures with repeated face-to-face, direct measures. For some phenotypes, self-report measures are gold standard (e.g., mental health)^10^ while for others (such as height and weight), self- or proxy-reported measures align closely with objective measurement.^11,12^ However, many childhood phenotypic precursors to adult NCDs are not routinely measured in clinical care and are asymptomatic (and therefore not known to parents or children). Earlier cohort studies had limited access to sophisticated measurement tools, and therefore focused on easy-to-measure phenotypes such as blood pressure, height and weight. With technological advances, today’s cohort studies can now directly measure multiple phenotypes across multiple body systems, in depth and at scale. These advances in measurement capabilities provide opportunities to construct life course trajectories of phenotypes relevant to major adult NCDs and characterize their variations, timing and drivers.

While the etiologies of many adult NCDs are now reasonably understood, it is less clear how to effectively act to alter their etiologic pathways and estimate the potential benefits of doing so. Mega-cohorts such as UK Biobank (*N* = 500,000) have demonstrated that size is critical in detecting patterns,^13^ with sample sizes of approximately 10,000 needed to build accurate prediction models.^14,15^ However, cohort studies for children with these large sample sizes are rare. Furthermore, few (if any) cohort studies have the repeated measures needed to construct phenotype trajectories across the entire life course and, even if they did, the early life components would reflect circumstances from many decades ago. Increasingly, researchers are seeking to combine data from multiple cohort studies to answer complex life course research questions. Retrospective harmonization of data can be challenging due to measurement heterogeneity, particularly for self-reported survey data or measures of latent constructs because of differences in construct definitions.^16,17^ However, direct measures of physiological phenotypes are typically measured as single summary values that can be standardized as z-scores and harmonized more readily than survey data.^18^ Methods and tools have been developed, for example, to combine phenotype measurements from multiple cohort studies across different, but overlapping, age ranges to piece together trajectories and characterize their patterns across the life course.^19^ Collaborations among cohort studies offer the opportunity to leverage the rich datasets available and solve these public health challenges.^20^

To capitalize on existing resources and inform planning for new cohort studies, an understanding is needed of the NCD-relevant phenotypic data available from recently established, large-scale cohorts. Therefore, we seek to identify contemporary, large (*N* > 8000) children’s longitudinal cohorts to map their direct phenotype measurement. This will identify both strengths and gaps in life course measurement internationally that may be critical to understanding NCD development and later burden of disease. An understanding of measurement gaps in areas relevant to major NCDs could inform the design of future data collection waves, to prioritize areas in need of more longitudinal research. This could also guide future data harmonization and cross-cohort collaboration. Collaboration could bridge gaps in individual cohorts with the same measures at different ages, increase statistical power and precision through pooling, and characterize differences or similarities in trajectory patterns across different populations, geographic locations and socioeconomic contexts. These efforts will enable researchers to use the rich datasets that cohort studies have developed to solve cutting-edge questions and to drive change in public health policies and practice that is urgently needed to address the growing burden of NCDs.

## Aims

The aims of this scoping review were to (1) identify large-scale, contemporary, population-based cohort studies that measure phenotypes relevant to major adult NCDs from childhood; (2) synthesize NCD-relevant phenotypes into a set of domains consistent across studies; and (3) provide a comprehensive overview of phenotype and domain measurement across studies, including the ages at which they are measured and the methods used to measure them. Through addressing these aims, we also intended to identify potential opportunities for data harmonization and collaboration across cohorts, and to identify gaps in the measurement of important health domains relevant to major NCDs.

## Methods

This scoping review was conducted following the Joanna Briggs Institute (JBI) methodology for scoping reviews,^21^ with minor modifications to the screening process where appropriate. As recommended by the JBI, our scoping review follows the Preferred Reporting Items for Systematic Review and Meta-Analyses, Scoping Review extension (PRISMA-ScR) guidelines (see Supplementary Material S1 for PRISMA-ScR checklist).^22^ Scoping reviews are appropriate for addressing exploratory research questions where the aims are to synthesize evidence across a large body of literature, identify coverage, or to provide an overview of concept definitions.^23^ Systematic reviews are better suited to addressing specific research questions, for example about treatment efficacy.^24^ We chose to conduct a scoping review, rather than a systematic review, because it was the most appropriate review type to address our aims of mapping the phenotypes measured by cohort studies and identifying gaps. We pre-specified our scoping review’s aims, eligibility criteria and methodology in our protocol at https://osf.io/7a8hd.

### Eligibility criteria

#### Participants

We included cohort studies that recruit participants in the pregnancy, perinatal or childhood periods, before age 20 years (the age when adulthood begins, as defined by the World Health Organization).^25^ We included cohort studies that draw participants from the general population (i.e., recruitment is not restricted to specific conditions or circumstances).

#### Concept

We considered cohort studies that directly measure (as opposed to self- or proxy-report) phenotypes relevant to the top 10 noncommunicable causes of global disease burden in adults aged 75 and above: cardiovascular diseases (ischaemic heart disease, stroke, hypertensive heart disease), COPD, Alzheimer’s disease, diabetes, accidental falls, chronic kidney disease, age-related hearing loss, and blindness and vision loss.^2^ We included falls (an injury) due to the life course influences of bone density and muscle strength on the likelihood of falls occurring and resulting in fracture.^26^ We excluded cancer both because it has a large stochastic component (i.e., begins with a random mutation) and, although many risk factors operate across the life course, clear intermediary phenotypes are not evident.^27^ We also excluded low back pain because it is usually episodic rather than progressive,^28^ and evidence is unclear regarding intermediary phenotypes that directly lead to it.^29^

#### Context

We included individual, recently-commencing cohort studies with a baseline sample of ≥8000 child participants. Although evidence suggests sample sizes >10,000 are needed to detect patterns and develop accurate risk prediction models for NCD-relevant phenotypes,^14,15^ a preliminary literature review indicated that limiting studies to those with sample sizes >10,000 would yield a small number of studies for inclusion, while >8000 identifies important additional studies. We considered cohort studies that recruit participants on an individual level (i.e., not household-level surveillance studies) and that cease recruiting during or after 2010.

### Search strategy

Usually, searches are intended to identify individual studies reported in a single publication. However, cohort studies are specifically intended to generate multiple publications. Our search strategy was therefore designed to identify relevant cohort studies from which we could then identify relevant sources for data extraction. We ran database searches on 03 September 2024 in MEDLINE, Embase and PubMed (see Supplementary Material S2 for search strategy) that included the terms cohort ‘profile’ or ‘protocol’, which are publications that describe the methodology and participants of cohort studies. We also searched websites and study networks that list or catalogue cohort studies (Supplementary Material S2). We limited inclusion to cohort studies with publications in English, due to a lack of resources to translate materials; however, recruitment could be in any or many languages.

### Study screening

Citations identified from the database search were uploaded to Covidence systematic review software^30^ and duplicates were removed using the in-built duplicate-removal function. Records were then dual-screened for inclusion by title and abstract. Following this, two authors independently reviewed all potentially relevant texts in full. Disagreements were resolved through discussion between the two reviewers and, if an agreement could not be reached, were resolved through discussion with the wider research team (i.e., authors of this paper).

Websites and study networks were manually reviewed by one author, following a pilot where two authors reviewed two websites independently to check for consensus. Eligible cohort studies identified from websites and study networks were listed in an Excel document.

### Data extraction

We developed a data extraction form in Qualtrics.^31^ The data extraction form was piloted and iteratively revised. As cohort studies often have many associated publications and materials from which to extract relevant information, data was extracted from multiple sources (see Supplementary Material S3). Where available, data extraction was prioritized from published cohort protocols and profiles. Individual cohort study websites were also important resources for data extraction, particularly if the website contained data collection protocols, a data dictionary, or described the measurements collected. If these sources did not yield sufficient evidence, relevant publications were identified through a Google Scholar search for publications that used data from the cohort study and described the data collection methods. If information was still unclear, we contacted the cohort study directly for clarification.

The data extracted included the study aim(s) and focus, recruitment details, sampling frame, sample size, study location, data access, and phenotype measurement (including the specific phenotype, participant age at measurement and methods used). We extracted information on the measurement of phenotypes relevant to our NCDs of interest defined above (see ‘Concept’ section). We extracted direct measurements only, except for measures of body size or composition, where both direct and reported measures were extracted. This was done because self- and parent-reported measures of body size or composition (such as height and weight) have been shown to have a high level of accuracy.^11,12^

### Evidence synthesis

The characteristics of each cohort study, including the location, focus, sampling frame, recruitment details, sample size, data collection, and data access were summarized. We synthesized phenotypes into nine overarching domains, with domains corresponding to body systems relevant to our NCDs of interest (see ‘Concept’ section). The domains included cardiovascular health (relevant to cardiovascular diseases), pulmonary function (COPD), the musculoskeletal system (falls), vision (blindness and vision loss), neurocognition (Alzheimer’s disease), kidney function (chronic kidney disease), hearing (age-related hearing loss), glucose control (diabetes) and body size or composition (relevant to most NCDs). The domains measured in each of the cohorts across childhood were summarized in a figure, to provide a domain-level overview of coverage and gaps. Additionally, we created an interactive, searchable table with more detail on the measurement of specific phenotypes using the Shiny package in R 4.3.0.^32,33^

## Results

### Search and study characteristics

**Fig. 1:**
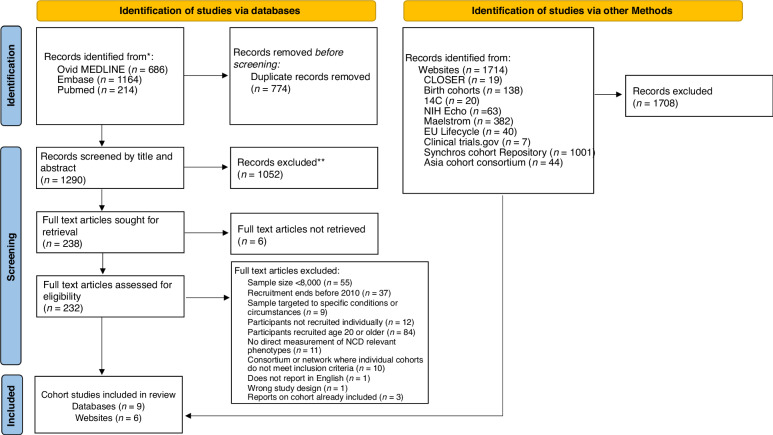
PRISMA flow chart. Source: Page MJ, et al. BMJ 2021;372:n71. 10.1136/bmj.n71.

The database search identified 214 articles in PubMed, 686 in Ovid MEDLINE and 1164 in Embase. After removing duplicate records, we screened 1290 articles by title and abstract and excluded 1058 articles. We then reviewed the full texts of the remaining 232 articles and identified 9 cohort studies for inclusion in this review. Our review of websites and study networks identified 1714 records. Duplicates could not be automatically removed and were instead removed manually during the screening process. We excluded 1708 records and identified an additional 6 unique cohort studies. Figure 1 shows the study screening and selection process.

Table 1 lists the cohorts and summarizes the characteristics of the identified large children’s life course phenotypic cohorts. Their international rarity is immediately evident. We identified only 15 such cohorts recruiting since 2010,^34–48^ of which only seven commenced in the last decade. Most of the included cohort studies are birth cohorts (*n* = 9), recruiting participants either during pregnancy or soon after birth. Most of the cohort studies are located in high-income countries (*n* = 11), with three based in Japan and one based in each of the USA, UK, France, Finland, Australia, South Korea, the Netherlands and Norway.^49^ One study is based in Iran (lower middle income) and the remaining three are based in China (upper middle income).^49^ The baseline sample sizes of the included cohort studies ranged from 8066 (Young-HUNT4) to 100,148 (Japan Environment and Children’s Study) child participants.

**Table 1:** Cohort study characteristics.

Cohort study and country	Focus	Sampling frame	Recruitment period	Age at recruitment	Sample size (baseline)	Data collection schedule
Adolescent Brain Cognitive Development (ABCD) Study; USA	Brain development and child health.	Probability sampling of schools within 21 catchment areas. Oversampling of schools with a student body >10% African American and located in rural/non-urban areas.	Sep 2016–Aug 2018	9–10 years	11,875 children	In-person assessments once a year, brief remote assessments at 6 months between in-person assessments. Yearly self-report, behavioral and biospecimen collections. Bi-annual brain imaging. Parents complete annual in-person iPad tasks and interviews, and 3- to 6-monthly phone interviews. Plan to follow up for 10 years (until age 19 – 20).
Born in Bradford (BiB); UK	Impacts of genetic, nutritional, environmental and social factors on health and development in childhood.	Eligible population was all pregnant women who planned to and gave birth at the Bradford Royal Infirmary (the only maternity unit in Bradford) during the recruitment period, their live born offspring and their partners.	Mar 2007–Dec 2010	Pregnancy (26–28 weeks gestation)	13,776 children	Whole cohort: Health worker visit at 2 weeks, 7 weeks and 8 months after birth. Primary school nurse data collection at age 5 – 6. Multi-method approach (community-based family visits, school-based physical assessment, whole classroom measures) at age 6 – 11. Sub-cohorts: variable.
Born in Guangzhou Cohort Study (BIGCS); China	The role of social, biological and environmental influences on pregnancy and child health and development.	Eligible population included all women of Chinese nationality, living in Guangzhou, at 20 weeks of gestation and intend to deliver at one of the two Guangzhou Women and Children’s Medical Center campuses.	Feb 2012–Dec 2023	Pregnancy	33,000 children	Neonatal birth information obtained from routine medical records. Interviews with mothers and child examinations in child health clinics at age 6 weeks, 6 months, 12 months and 36 months, or by phone. Plan to follow up all children until age 18 years.
Chengdu Positive Child Development (CPCD) survey	Associations between school students’ sociodemographic, physical and psychological characteristics, family environment, lifestyle behaviors, academic performance and health status.	Cluster sampling to select five primary and middle schools in Chengdu from which students were recruited.	Dec 2019–Jan 2020	6–16 years	8825 children	Baseline survey completed by students’ caregivers and school principals. First follow-up conducted 6 months after baseline to capture the immediate impacts of the coronavirus disease 2019 (COVID-19). Future follow ups planned via data linkage and questionnaires.
The French National Cohort of Children (ELFE); France	The relationship between environmental exposures and socio-economic factors on health and behavior.	Randomly selected 349 maternity units in metropolitan France, of which 320 participated. Stratified sampling strategy based on size of each unit to allow for oversampling in larger units. Recruitment took place during 25 selected days, grouped into four periods over the year. 51% of contacted women agreed to participate.	2011–2011	Birth	18,040 children	Interview and biosample collection at maternity unit after birth. Data collection waves take place approximately every 1 – 2 years and can include phone interviews, questionnaires, questionnaires for family or nursery school doctor, home visit, preschool teacher survey, web game questionnaires. Aim to continue follow up until children are age 20 years.
The Finnish Health in Teens (FIN-HIT) Study; Finland	Obesity, weight, weight gain and weight-related health outcomes.	Pilot: Home-based recruitment by mailed invitation among 11,000 randomly selected households. Participation rate at baseline of 30%. Main: School-based recruitment in which 496 schools throughout Finland agreed to participate. Fieldworkers handed out invitations to 27,000 adolescents. Participation rate at baseline of 36%.	Pilot: 2011–2011 Main: 2013–2014	9–12 years	11,407 children	First active follow-up at age 13–15 years. Second active follow up at age 18–23 years (underway). Web questionnaires once participants reach 18 years. Participants have consented to link national health register data. Current ethical approval for follow-up until age 25 years.
Generation Victoria (GenV); Australia	Physical health, mental health and social issues during childhood. Childhood and adult antecedents of health or disease during ageing.	Eligible participants include all individuals born in a 2-year period and living in the state of Victoria, and their parents.	Advance birth window: Dec 2020–Oct 2023 Statewide birth window: Oct 2021–Oct 2023	Birth for establishment phase; 'Door’s Always Open’ recruitment at any age and indefinitely if in birth window	49,217 children at time of submission	Survey questions and digital assessments via web and smartphone app sent every 3 months until age 12 months, then every 6 months. Planning face-to-face phenotypic waves at 6, 11 and 16 years.
Hokkaido Birth Cohort Study on Environment and Children’s Health; Japan	Effects of environmental exposure and genetic predisposition on health and development in the prenatal period, infancy and early childhood.	Hokkaido: Eligible participants included pregnant women that visited one of the 37 hospitals or clinics in the Hokkaido area for prenatal healthcare. Sapporo: Eligible participants included pregnant women who delivered at Toho hospital.	Hokkaido: Feb 2003–Mar 2012 Sapporo: Jul 2002–Oct 2005	Pregnancy (Hokkaido: before 13 weeks gestation; Sapporo: 23–25 weeks gestation)	Hokkaido large-scale: 20,926 pregnant women Sapporo: 514 pregnant women	Survey at baseline and follow up surveys at child age 1, 2, 4, 5, 6, 7, 8 and 12 years. Face-to-face examinations in the Sapporo cohort at child age 6–7 months, 1.5, 3.5, 7 and 11–14 years. Face-to-face examinations in the Hokkaido cohort at child age 7, 9–11, 11–14 and 14–17 years. School-based data collection at age 13 in both cohorts. Additional data and biosample collection in Hokkaido cohort at age 8, 9–11 and 12 years.
Japan Environment and Children’s Study (JECS); Japan	The impact of environmental factors on children’s health and development.	Eligible participants included pregnant women recruited from prenatal healthcare examinations and local government offices in one of 15 Regional Centers. Regional centers were selected in a competitive process in which universities and research institutes submitted proposals.	Jan 2011–Mar 2014	Pregnancy	Main: 100,148 Sub-cohort: 10,302 children	Questionnaire and biosample collection at baseline. Biosample collection and baby health check at birth. Questionnaires every 6 months and face-to-face examination every several years. Environmental measurement from child age 6 months to 13 years. More in-depth follow-up in sub-cohort.
The Korean Children’s Environmental Health Study (Ko-Chens); South Korea	The effects of environmental exposures on health and environment-related diseases in children.	Eligible participants included pregnant women recruited from one of 14 centers (one coordinating center, one supportive center, and 12 regional centers).	Main: 2015–2019 Core: 2015–2018	Pregnancy	Main: 65,000 Core: 5,000 children	Main Cohort: Questionnaires and biosample collections at baseline and each follow-up. Follow-ups every year from birth to age 7 years and several school-based follow-ups up to 1st grade middle school.Core Cohort: More detailed baseline questionnaire. Follow up at age 6 months, every year from age 1 to 7 years, school-based follow up at age 7, 10, 13 and 16 years.
Lifelines; Netherlands	The etiology of healthy ageing.	Eligible participants included adults aged 25–50 years who were invited to participate through their General Practitioner (GP). GPs were based within three northern provinces of The Netherlands (Friesland, Groningen and Drenthe). Participants gave permission for their family members to be invited to participate.	2006–Dec 2013	Children are recruited at any age 0–18 years	14,801 children	Questionnaire every 1.5 years. Participants are invited to an in-person physical examination approximately every 5 years. Participants aged ≥8 years were invited to a face-to-face baseline assessment for physical examination. Linkage with health records and national registries.
The Prospective Epidemiological Research Studies in Iran (PERSIAN) Birth Cohort; Iran	The role of socio-economic factors, lifestyle, diet, environmental exposures and epigenetic factors with mother and child health outcomes.	Eligible participants included pregnant women with Iranian nationality who have resided in the catchment area of the study center for at least one year, planned to give birth in a hospital located in the study area and plan to live in that city for the next 5 years. Enrollment took place in five Iranian cities (Isfahan, Yazd, Semnan, Rafsanjan and Sari).	Oct 2016–not described	Pregnancy	15,000 children (planned)	Child follow-up sessions take place at health centers at the time of routine child vaccinations at age 2, 4, 6 and 12 months. Annual follow-ups from age 24 months. The study provides free health check-up visits at pediatrician clinics every 4–6 months. Follow-ups include questionnaires, biosamples, physical examinations, clinical tests, hospital records, household survey and linked geocoded data. Plan to continue follow up until age 11 years.
The Shanghai Children’s Health, Education and Lifestyle Evaluation, Preschool (SCHEDULE-P) Study; China	Early childhood development.	Selected a representative sample from newly enrolled children in the kindergarten registration system of the Shanghai Municipal Education Commission.	Sep 2016–not described	3–4 years	20,899 children	Three waves of online survey in 2016, 2018 and 2019. Field survey in 2019.
Tohoku Medical Megabank Project Birth and Three-Generation Cohort Study (TMM BirThree Cohort Study); Japan	Common diseases that involve the interplay between genetic and environmental factors and the aftermath of the Great East Japan Earthquake.	Pregnant women and their fetuses were recruited from approximately 50 obstetric clinics and hospitals in the Miyagi Prefecture. The women’s partners, parents, children, and extended family members were also invited to participate, regardless of genetic relationship.	Jul 2013–Mar 2017	Pregnancy and siblings of newborn <20 years	32,602 children	Questionnaires are mailed to parents when children are age 6, 12, 24, 36, 42, 48 and 60 months. Questionnaires are sent once per year after the child reaches age 5 years. Linkage to municipality-based health examination, school health examination records and other systems. Guardians are asked to take their children to community support centers for health assessments at approximately age 4, 10 and 16 years, and bring the children’s maternal and child health handbooks for transcription.
Young-HUNT4 Study; Norway	Major public health issues including diseases, health behaviors, and mental health.	Eligible participants included all residents aged 13–19 years in the northern part of Trøndelag Country. 76% participation rate from those invited.	2017–2019	13–19 years	8066 children	First wave conducted in 2017–2019 where participants completed a questionnaire on electronic tablets, participated in clinical measurement and interview, and saliva samples were collected. Most adolescents participated while at school. Previous Young-HUNT cohorts have been followed up in later HUNT (adult) waves.

### Measurement of NCD-relevant phenotypes and domains

**Fig. 2:**
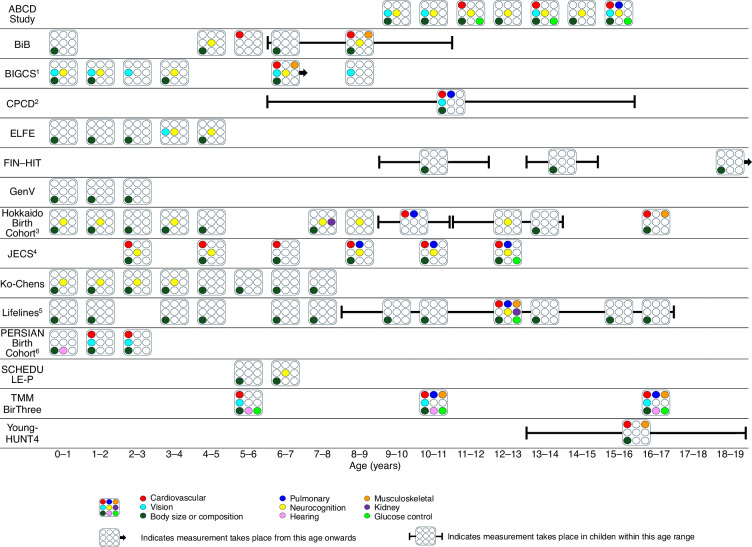
Phenotype domains measured by cohort studies across age. ^1^BIGCS measures vision at precise ages. The vision measurement at age 6 years is standalone, unlike the other domains at that age which are measured in children >6 years of age. ^2^CPCD plans to repeat measures at annual school physical examinations. ^3^The Hokkaido Birth Cohort comprises two separate birth cohorts with some differences in the phenotypes and ages measured in each. See Shiny app for more details. ^4^Some measures in JECS are conducted in the sub-cohort only. See Shiny app for more details. The measures at age 10 and 12 years are planned. ^5^In Lifelines, some phenotypes are measured at the baseline assessment and are repeated at the second assessment, whereas others are only measured in one assessment. See Shiny app for more details. ^6^The PERSIAN birth cohort plans to measure neurocognition but has not specified the age(s) when this will be measured. It is therefore missing from this figure, but is included in Table 2 and in the Shiny app. In addition to directly measuring body size or composition at the ages shown on this figure, body size or composition is also collected every 2 months from the participant’s logbook, and no end date has been specified.

Figure 2 summarizes the domain-level phenotype measurements in each cohort study against age, and Table 2 summarizes their specific phenotypes measured. A more detailed overview of these phenotypes within each domain, including the age(s) at which they are measured, the measurement tools used and the location of data collection, is provided in an interactive, searchable table accessible via this link: https://katiemcbain.shinyapps.io/Cohortmeasures/.

**Table 2:** Phenotypes measured by cohorts within each domain (see Shiny app for measures detail).

	ABCD	BiB	BIGCS	CPCD	ELFE	FIN-HIT	GenV	Hokkaido Birth Cohort	JECS	Ko-Chens	Lifelines	PERSIAN Birth Cohort	SCHEDULE-P	TMM BirThree	Young-HUNT4
BODY SIZE OR COMPOSITION															
Anthropometrics	**●**	**●**	**●**	**●**	**●**	**●**	**●**	**●**	**●**	**●**	**●**	**●**	**●**	**●**	**●**
Body size or composition (NS)														**●**	
Bioimpedance body fat		**●**	**●**						**●**						**●**
DEXA body fat		**●**													
NEUROCOGNITION															
Brain imaging	**●**														
Cognition	**●**	**●**	**●**		**●**			**●**	**●**		**●**		**●**		
Event-related potentials								**●**							
Neurodevelopment			**●**		**●**			**●**	**●**	**●**		**●**			
CARDIOVASCULAR															
Blood pressure	**●**	**●**	**●**	**●**				**●**	**●**		**●**	**●**		**●**	**●**
Heart rate variability											**●**			**●**	
Pulse rate		**●**		**●**					**●**		**●**				**●**
MUSCULOSKELETAL															
Bioimpedance muscle		**●**													**●**
Calcaneal ultrasound														**●**	
DEXA bone density		**●**	**●**												
DEXA muscle mass		**●**													
Grip strength			**●**					**●**						**●**	
Strength jump test											**●**				
GLUCOSE CONTROL															
Glucose									**●**		**●**				
Glycoalbumin														**●**	
HbA1c	**●**								**●**		**●**			**●**	
Insulin									**●**						
PULMONARY															
FeNO								**●**	**●**						
Spirometry									**●**		**●**				
Lung function (NS)	**●**													**●**	
Vital capacity				**●**											
VISION															
Axial length														**●**	
Eye refraction												**●**		**●**	
Visual acuity	**●**			**●**	**●**									**●**	
Vision screening			**●**												
HEARING															
Auditory test												**●**			
Hearing acuity														**●**	
KIDNEY															
Blood creatinine								**●**			**●**				
Blood ureum											**●**				
Blood uric acid											**●**				
Urine albumin											**●**				
Urine creatinine											**●**				

*NS* not specified.

#### Cardiovascular

Cardiovascular health is measured by ten of the 15 cohort studies on at least one occasion. Measurement spans from age 1–19 years, with a gap at age 3-4. All ten of these studies measure blood pressure, while five also measure pulse rate and two measure heart rate variability using an electrocardiogram.

#### Pulmonary

Six of the cohort studies measure pulmonary function, but only two measure it repeatedly. Collectively, pulmonary function is measured from age 6–17 years. Two of the cohort studies measure lung function using spirometry, one measures vital capacity instrumentally, two assess airway inflammation using exhaled nitric oxide (FeNO) and two measured lung or respiratory function but did not specify the measurement tool used.

#### Musculoskeletal

Musculoskeletal phenotypes are measured by six cohort studies, collectively from age 6 years. Specific musculoskeletal phenotypes measured by the cohort studies include bone density, which is measured using dual x-ray absorptiometry (DEXA) by two studies and calcaneal ultrasound imaging by one study; muscle mass, which is measured using DEXA by one study and bioimpedance by two studies; strength, which is measured using a jump test by one study; and grip strength, which is measured by three studies.

#### Vision

Vision is measured by six cohort studies, collectively from the first year of life to age 16 years. Within this domain, visual acuity is measured by four studies, eye refraction by two studies and axial length by one study. One study describes assessing vision through vision screening but did not provide details on the exact measures used.

#### Neurocognition

Nine of the cohorts have measured neurocognition at least once. Together, cohorts that measure neurocognition assess this domain each year from the first year of life to age 17 years, excluding age 5–6 years. Cohort studies measuring neurocognition use a wide range of measures that assess many different phenotypes, with very few occasions of the same measure being used in multiple cohort studies; in total, we identified 53 unique measures in this domain. Of the six cohort studies that measured the subcategory of neurodevelopment, two use the Ages and Stages Questionnaire, two use the Denver Developmental Screening Test, and two use the Bayley Scales of Infant Development. Of the three cohort studies that measure intelligence, all use the Weschler Intelligence Scale for Children, alongside other measures.

#### Kidney

Two of the cohort studies measure kidney function, with one study measuring it on two occasions in children aged between 8 and 17 years and the other measuring it at age 7 years. One study measures kidney function through measures extracted from urine samples (albumin and creatinine) and blood (urea and uric acid). Both studies extract measures of creatinine from blood samples (enabling estimation of glomerular filtration rate, eGFR).

#### Body size or composition

All 15 cohort studies measure body size or composition on at least one occasion, and all but two do so repeatedly. Collectively, the cohort studies measure body size or composition every year from birth to age 19 years. Specific phenotypes measured within this domain vary and include anthropometric measurements (e.g., weight, height, waist circumference, arm circumference, skinfold thickness), bioimpedance and DEXA body scans. Of the five cohort studies that include self- or parent-reported anthropometric measurements, three of them also include direct, in-person measurements.

#### Hearing

Hearing is measured by two of the cohort studies, spanning ages 0-1, 5-6, 10-11 and 16-17 years. Hearing is measured using an auditory test by one study, and the other study states it measures hearing acuity but not the measure used.

#### Glucose control

Glucose control is measured by four cohort studies, collectively spanning age 5, 6 and 8–17 years. Of the four studies that measure phenotypes in this domain, all four measure HbA1c, two measure blood glucose, one measures blood insulin and one measures blood glycoalbumin.

### Summary of measurement coverage and gaps

**Fig. 3:**
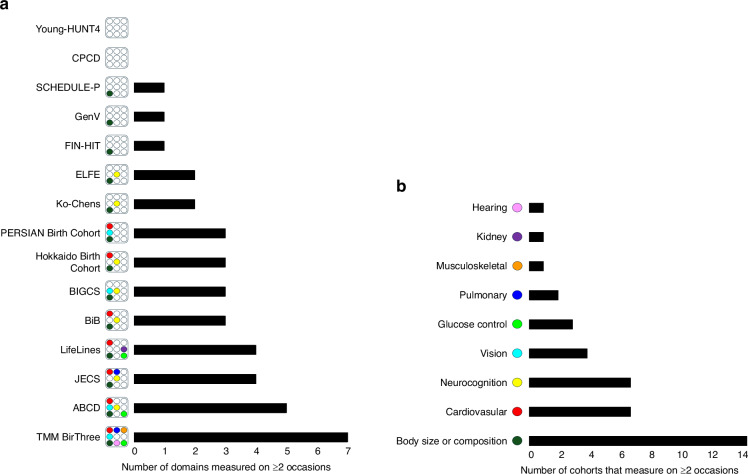
Repeated measurement across domains and cohort studies. **a** Cohort studies that repeat measurement, ranked from least to most domains repeated. **b** Domains measured on at least two occasions, ranked from least to most repeated domains.

The health domains measured by the most cohort studies are body size or composition, cardiovascular health and neurocognition. These are also the domains with the greatest number of studies measuring them on at least two occasions (Fig. 3). Measurement across the cohort studies also spans the longest childhood age range for these three domains. The most diversity of phenotype measures is in the neurocognition domain, which may limit cross-cohort analyses.

The health domains measured by the fewest number of cohort studies are hearing and kidney function. These are also the domains with the fewest number of cohort studies repeating measurement on at least one other occasion, alongside the musculoskeletal domain.

### Data access across cohorts

All of the included cohort studies describe how data can be accessed. Only one specifically describes being Open Access. Details on data access for each study are summarized in Table 3.

**Table 3:** Data access across the cohort studies.

Cohort	Data access details	Contact/Requests
ABCD	ABCD is an Open Science study. Data are released annually. Access must be approved by the NDA Data Access Committee.	Complete the Data User Certificate for access. Visit [https://nda.nih.gov/study.html?id=2147].
BiB	Data can be accessed after request is approved and Data Sharing Contract and Data Sharing Agreement are signed. There is a fee for accessing data.	Submit EOI form via email [borninbradford@bthft.nhs.uk].
BIGCS	Data access will begin when >50,000 pregnant women have been recruited and their children reach at least 3 years old. Data use must obey relevant laws and regulations of People's Republic of China. All projects must be approved by the Committee of Ethics. Approved projects will be considered as sub-projects of BIGCS.	Submit an application to the Guangzhou Women and Children Medical Center via email [data.bigcs@bigcs.org].
CPCD	Data may be shared on reasonable request.	Contact the principal investigator Professor Peng Jia at [jiapengff@hotmail.com].
ELFE	Data can be accessed by any public or private sector research team providing it meets the conditions set out in the data access charter. Exclusive access given to research teams who helped to design ELFE for the first 18 months, after which access is extended to the whole scientific community. Members of research teams not affiliated with ELFE must have their project approved by the Committee for Access to Elfe Data and submit a commitment to comply with methodological guidelines to the French Data Protection Authority.	Requests for access can be made via Pandora, a secure internet platform.
FIN-HIT	FIN-HIT welcomes collaborative research partners.	Requests can be sent to Principal Investigator Heli Viljakainen via email [heli.viljakainen@helsinki.fi] or phone [+358 50 4485660].
GenV	Currently developing data and biosample access frameworks. GenV’s data is intended to be equal access, via the FAIR and Five Safes principles, with limited periods of protected data access from the point at which a complete usable dataset is available.	Sign up for updates on data access via the website [https://www.genv.org.au/for-researchers/inquire-about-the-data/].
Hokkaido Birth Cohort	Data are not currently publicly available but can be made available upon request.	Email corresponding author–[rkishi@med.hokudai.ac.jp] is the corresponding author on the most recent (2021) cohort protocol.
JECS	Data are not currently publicly available due to ethical restrictions and legal framework of Japan.	Inquiries about data access can be sent via email to [jecs-en@nies.go.jp]. The person responsible for handling enquiries sent to this e-mail address is Dr Shoji F. Nakayama, JECS Program Office, National Institute for Environmental Studies.
Ko-Chens	Limited access to data stored on the KoCHENS Database System.	
LifeLines	Researchers from public institutes can apply by submitting a proposal to the LifeLines Research Office. Proposals are reviewed by the LifeLines scientific board. There are costs associated with accessing data and biomaterials.	Submit proposal to the LifeLines Research Office [LLscience@umcg.nl].
PERSIAN Birth Cohort	Plan to develop a comprehensive data catalogue in the future with plans to participate in collaborations. Decisions on data sharing will be made by the cohort steering and scientific committees.	
SCHEDULE-P	De-identified data can be shared for research purposes. Data are held and managed by the research group at Shanghai Children’s Medical Center. Access via application which is approved by the Publication Committee.	Contact Dr Fan Jiang [fanjiang@shsmu.edu.cn] and Dr Yunting Zhang [edwinazhang@hotmail.com] to discuss potential collaboration.
TMM BirThree	Currently, Japanese research institutions have full access to data. Researchers in countries outside Japan can access phenotypic data and summary statistics. Currently discussing international access to genetic data.	
Young-HUNT4	Research groups with a principal investigator affiliated with a Norwegian research institution may apply for data access. Agreements are made between research institutions and the Faculty of Medicine, Norwegian University of Technology and Science, Trondheim. Applications must be approved by the Regional Committee for Medical and Health Research Ethics in Mid-Norway.	Applications can be sent to HUNT Research Center. Information on the HUNT website [http://www.ntnu.edu/hunt.Enquiries] or contact Professor Turid Lingaas Holmen, HUNT Research Center [turid.lingaas holmen@ntnu.no] or Kirsti Kvaløy at [kontakt@hunt.ntnu.no].

## Discussion

### Principal findings

This is the first scoping review to provide a comprehensive overview of phenotype and domain measurement in large, population-based child cohort studies. We identified 15 cohort studies that directly measure phenotypes relevant to major adult NCDs from childhood. Body size or composition is the most widely measured domain across the cohort studies, closely followed by cardiovascular health and neurocognition. Less than half of the cohort studies measure phenotypes within the domains of musculoskeletal function, pulmonary function, vision and glucose control. Hearing and kidney function are measured by only two cohort studies. Phenotypes measured across several cohorts offer opportunities for collaboration and data harmonization, spanning multiple years in childhood. Our review has identified gaps in the measurement of other important phenotypes, which limit opportunities to map their trajectories and to address research questions targeting these phenotypes and their relevant NCDs. Findings from this review may assist in the planning and design of future cohort studies and analyses.

### Strengths and limitations

A strength of this scoping review is that we not only searched for eligible cohort studies via publications on databases, but we also searched gray literature in the form of websites (including study registration sites) and study networks. This enabled us to find cohort studies that have not yet published cohort protocols or profiles, particularly newer studies that are not yet at the stage of sharing these publications, such as GenV. By focusing on major adult NCDs that contribute to global disease burden, we have highlighted the phenotypic data available in childhood to address major population challenges. Our interactive, searchable Shiny app table, allows researchers and other stakeholders interested in these data to readily search for and identify cohort studies with relevant measurements, facilitating collaboration or the wisdom of plugging gaps at the earliest opportunity.

This scoping review also has limitations. Some cohort studies may not have yet publicly shared details of all data collection waves, and others may have changed plans shared in earlier publications. Researchers seeking to use the data described in this review should verify details through direct contact with the relevant cohort studies and reviewing technical documents where available in data access portals. Large cohort studies are expensive to run and are funded for short periods at a time, meaning that data collection is planned in stages, focusing on funded age ranges. Because we reviewed contemporary studies, some of the newer studies recruiting participants at birth (such as GenV) are only beginning to collect measurements early in the life course, whereas other studies (such as the ABCD Study) have already collected measures in older age ranges. The most widely measured health domains in the older childhood years, and the most often repeated measures, may shift over time as younger participants in newer cohort studies reach those later ages.

### Interpretation of findings and implications

It is not surprising that the most widely-measured phenotypes across these cohort studies fall within the domains of body size or composition, cardiovascular health and neurocognition. These domains are relevant to later life conditions with high burden of disease – cardiovascular disease is the leading cause of global disease burden from age 50 years onwards, while Alzheimer’s disease is the fourth highest contributor in adults 75 and over.^2^ Childhood overweight and obesity is a major public health challenge in many countries and is a recognized risk factor for many adult NCDs.^50^ These three health domains can be measured using a variety of tools, including those that are fast, non-invasive and affordable, such as body mass index via anthropometry or blood pressure via sphygmomanometer. These measures are well suited to large-scale, population-based research, and were the dominant measures used across the cohort studies in this review. Other important measures of these domains that are invasive, costly, time-consuming or bulky, such as carotid-artery intima-media thickness, were completely absent from these very large cohort studies. This highlights the importance of low-cost, low burden measures for these types of studies.

Where phenotypes are measured widely and at multiple ages throughout childhood, our findings suggest that there are opportunities for cross-cohort collaboration and data harmonization. Pooled data analysis could be undertaken, for example, to construct phenotype trajectories that include datapoints at most, if not all, of the childhood years for body size or composition, neurocognition and cardiovascular phenotypes. This could be done using methods that allow trajectories to be developed from multiple cohorts spanning different, but overlapping, age ranges, such as those developed by Hughes et al.^19^ As measures of body size or composition are regularly repeated across the cohort studies, trajectories could be compared across cohorts to investigate the role of contextual influences such as socioeconomic factors, environmental conditions, or policies.

We did not include umbrella initiatives bringing together multiple smaller cohorts. For example, the ~60,000 children in the USA-based Environmental influences on Child Health Outcomes (ECHO) Cohort^51^ come from 69 individual cohorts that vary in decade, geography and life stage at recruitment and who they represent (general population or at high risk of a targeted condition), with no single participating cohort individually meeting our pre-specified inclusion criteria. We fully acknowledge that ECHO’s standardized prospective measurement^52^ of heart rate, blood pressure, body composition and anthropometry, neurodevelopment and lung function (via spirometry) will further strengthen phenotypic data beyond that reported in our review.

While this scoping review has identified promising opportunities for collaboration and pooled analyses, we have also identified notable gaps in the measurement of health domains relevant to burden of disease. Cohort studies represent significant investments in population health and provide important opportunities to identify prevention strategies, but today’s large child cohorts are not capturing the precursors to some high-burden adult NCDs. This will limit our ability to understand early life trajectories to these important conditions of ageing. There may be several reasons for these measurement gaps, relating to the invasiveness, costs, or time involved in measuring some domains and phenotypes. Hearing can be challenging to measure, particularly in large cohort studies which aim to measure multiple aspects of health in often short timeframes. Pure tone audiometry, the gold standard for measuring hearing, can be expensive and time-consuming, and traditionally has required a sound-proof environment, all of which are limiting for large-scale studies.^53^ The sparse measurement of this domain provides a rationale for new measures to be developed that are better suited for large-scale, population-based research. Kidney function, along with glucose control, are measured through biospecimen collections (blood and urine). Many of the cohort studies included in this review collect and store bio-samples, including urine and blood, but we have only reported on those that described extracting measures of either kidney health or glucose control. The reported gaps in measurement in these domains may therefore reflect either a low priority or the high cost and logistics of bioassays to extract measures from collected and stored samples. Therefore, stored samples reflect a further opportunity, as funding becomes available, to redress some of these gaps.

### Future research

This review highlights opportunities for analyses combining data from multiple studies, so that these important resources can become knowledge as soon as possible. There are also opportunities for future research to integrate these phenotypic measurements (phenome) with other multi-omic techniques to strengthen causal inference and enhance understanding of NCD prevention, including through personalized preventive approaches.^54,55^ While we have focused on large cohorts, sub- or nested cohorts offer rich and immediate opportunities for detailed analyses such as bioassays that may later become available to larger cohorts as technology and costs allow. Future cohort studies, or cohort studies planning data collection waves, may also use our findings to guide their planning. This can enable collaboration or address some of the measurement gaps identified. Launching and planned cohorts have the opportunity to address gaps by drawing on new imaging, wearable and adaptive technologies.^56–58^

This scoping review focused on a range of organ-level physiological phenotypes within nine high-level domains relevant to major adult NCDs of ageing. Future reviews may focus on risk factors for adult NCDs (e.g., physical activity), measures relevant to different areas (e.g., child health conditions) or an area in greater detail and including, for example, smaller cohort studies that may have more concentrated aims. Future reviews may look at phenotypes measured in adult cohorts, particularly as midlife is another important transitional period for health trajectories.^59^ Future research is also needed to identify or create cost-effective, low-burden measures that are suitable for large-scale research. Currently, it appears that limitations in the measures available to large cohorts may limit the translational evidence these studies can generate and that is needed to reduce future disease burden.

### Conclusions

We have mapped the availability and gaps in phenotypes relevant to major adult NCDs measured by contemporary large-scale child cohort studies. The widely-measured domains of body size or composition, neurocognition, and cardiovascular health offer opportunities for data pooling and harmonization that could support larger sample sizes, comparison across cohorts, or bridge gaps in individual cohorts by combining the same measures at different ages. However, for some important domains phenotypes are lacking (notably kidney function and hearing). If these gaps are not addressed, the enormous investments in these cohort studies will not adequately inform understanding of pathways to substantive portions of disease burden. Cross-cohort planning to measure key phenotypes within underrepresented domains could address these gaps. This will ensure that all major NCD precursors are adequately represented over time for greatest future benefit.

## Supplementary information

Supplementary information

## Data Availability

All relevant data are included in this manuscript and its supplementary information files.
