# Supplementary material for: A scoping review of adult NCD-relevant phenotypes measured in today’s large child cohort studies
Source: Pediatr Res. 2025 Apr 12;98(6):2058–72. doi: 10.1038/s41390-025-04056-3 (PMC12811134; doi:10.1038/s41390-025-04056-3)
Supplement: Supplementary file 1 — Supplementary information [file 41390_2025_4056_MOESM1_ESM.pdf]

Supplementary material S1: Preferred Reporting Items for Systematic reviews and Meta-Analyses extension for Scoping Reviews (PRISMA-ScR) checklist

SECTION	ITEM	PRISMA-ScR CHECKLIST ITEM	REPORTED ON PAGE #
**TITLE**			
Title	1	Identify the report as a scoping review.	1
**ABSTRACT**			
Structured summary	2	Provide a structured summary that includes (as applicable): background, objectives, eligibility criteria, sources of evidence, charting methods, results, and conclusions that relate to the review questions and objectives.	2
**INTRODUCTION**			
Rationale	3	Describe the rationale for the review in the context of what is already known. Explain why the review questions/objectives lend themselves to a scoping review approach.	5-6
Objectives	4	Provide an explicit statement of the questions and objectives being addressed with reference to their key elements (e.g., population or participants, concepts, and context) or other relevant key elements used to conceptualize the review questions and/or objectives.	5
**METHODS**			
Protocol and registration	5	Indicate whether a review protocol exists; state if and where it can be accessed (e.g., a Web address); and if available, provide registration information, including the registration number.	6
Eligibility criteria	6	Specify characteristics of the sources of evidence used as eligibility criteria (e.g., years considered, language, and publication status), and provide a rationale.	6
Information sources*	7	Describe all information sources in the search (e.g., databases with dates of coverage and contact with authors to identify additional sources), as well as the date the most recent search was executed.	7
Search	8	Present the full electronic search strategy for at least 1 database, including any limits used, such that it could be repeated.	Supplementary material S2
Selection of sources of evidence†	9	State the process for selecting sources of evidence (i.e., screening and eligibility) included in the scoping review.	8
Data charting process‡	10	Describe the methods of charting data from the included sources of evidence (e.g., calibrated forms or forms that have been tested by the team before their use, and whether data charting was done independently or in duplicate) and any processes for obtaining and confirming data from investigators.	8
Data items	11	List and define all variables for which data were sought and any assumptions and simplifications made.	9
Critical appraisal of individual sources of evidence§	12	If done, provide a rationale for conducting a critical appraisal of included sources of evidence; describe the methods used and how this information was used in any data synthesis (if appropriate).	NA
Synthesis of results	13	Describe the methods of handling and summarizing the data that were charted.	9
**RESULTS**			
Selection of sources of evidence	14	Give numbers of sources of evidence screened, assessed for eligibility, and included in the review, with reasons	10

SECTION	ITEM	PRISMA-ScR CHECKLIST ITEM	REPORTED ON PAGE #
		for exclusions at each stage, ideally using a flow diagram.	
Characteristics of sources of evidence	15	For each source of evidence, present characteristics for which data were charted and provide the citations.	10
Critical appraisal within sources of evidence	16	If done, present data on critical appraisal of included sources of evidence (see item 12).	NA
Results of individual sources of evidence	17	For each included source of evidence, present the relevant data that were charted that relate to the review questions and objectives.	10
Synthesis of results	18	Summarize and/or present the charting results as they relate to the review questions and objectives.	10-13
**DISCUSSION**			
Summary of evidence	19	Summarize the main results (including an overview of concepts, themes, and types of evidence available), link to the review questions and objectives, and consider the relevance to key groups.	14
Limitations	20	Discuss the limitations of the scoping review process.	15
Conclusions	21	Provide a general interpretation of the results with respect to the review questions and objectives, as well as potential implications and/or next steps.	18
**FUNDING**			
Funding	22	Describe sources of funding for the included sources of evidence, as well as sources of funding for the scoping review. Describe the role of the funders of the scoping review.	27

JBI = Joanna Briggs Institute; PRISMA-ScR = Preferred Reporting Items for Systematic reviews and Meta-Analyses extension for Scoping Reviews.
* Where *sources of evidence* (see second footnote) are compiled from, such as bibliographic databases, social media platforms, and Web sites.
† A more inclusive/heterogeneous term used to account for the different types of evidence or data sources (e.g., quantitative and/or qualitative research, expert opinion, and policy documents) that may be eligible in a scoping review as opposed to only studies. This is not to be confused with *information sources* (see first footnote).
‡ The frameworks by Arksey and O'Malley (6) and Levac and colleagues (7) and the JBI guidance (4, 5) refer to the process of data extraction in a scoping review as data charting.
§ The process of systematically examining research evidence to assess its validity, results, and relevance before using it to inform a decision. This term is used for items 12 and 19 instead of "risk of bias" (which is more applicable to systematic reviews of interventions) to include and acknowledge the various sources of evidence that may be used in a scoping review (e.g., quantitative and/or qualitative research, expert opinion, and policy document).

From: Tricco AC, Lillie E, Zarin W, O'Brien KK, Colquhoun H, Levac D, et al. PRISMA Extension for Scoping Reviews (PRISMAScR): Checklist and Explanation. Ann Intern Med. 2018;169:467–473. doi: 10.7326/M18-0850.

Supplementary material S2: Search strategy. (A) Search strategy for databases. (B) Details of website search.

(A) Database search strategy 03 September 2024

MEDLINE	PubMed	Embase
1. (cohort-profile* or cohort-protocol*).tw,kf.<br><br>2. epidemiolog*.tw,kf,hw.<br><br>3. ep.fs.<br><br>4. 2 or 3<br><br>5. (health* or disease*).tw,kf,hw.<br><br>6. 1 and 4 and 5	1. "cohort-profile*"[Title/Abstract] OR "cohort-protocol*"[Title/Abstract]<br><br>2. "epidemiolog*"[Title/Abstract]<br><br>3. "health*"[Title/Abstract] OR "disease*"[Title/Abstract]<br><br>4. NOTNLM OR publisher[sb] OR inprocess[sb] OR pubmednotmedline[sb] OR indatareview[sb] OR pubstatusaheadofprint<br><br>5. #1 AND #2 AND #3 AND #4	1. (cohort-profile* or cohort-protocol*).tw,kf,dq.<br><br>2. (health* or disease*).tw,kf,hw,dq.<br><br>3. 1 and 2

(B) Website search

Website	Date searched
https://www.birthcohorts.net/	26/04/2023
https://clinicaltrials.gov/	14/03/2023
https://www.closer.ac.uk/	26/04/2023
https://epi.ncc.go.jp/en/acc/index.html#:~:text=Asia%20Cohort%20Consortium%20(ACC)%20is,of%20one%20million%20healthy%20population.	14/02/2023
https://lifecycle-project.eu/for-scientists/the-eu-child-cohort-network/	08/02/2023
https://www.maelstrom-research.org/network/cohorts.se	14/03/2023
https://www.mcri.edu.au/research/projects/international-childhood-cancer-cohort-consortium-i4c/i4c-consortium	15/02/2023
https://www.nih.gov/research-training/environmental-influences-child-health-outcomes-echo-program	28/02/2023
https://repository.synchros.eu/	07/03/2023

Supplementary material S3: Included cohort studies and sources of data extraction

Cohort	Citations
ABCD	1. Adolescent Brain Cognitive Development. (2023, May 19). ABCD Study. https://abcdstudy.org/<br>2. Barch DM, Albaugh MD, Avenevoli S, Chang L, Clark DB, Glantz MD, Hudziak JJ, Jernigan TL, Tapert SF, Yurgelun-Todd D, Alia-Klein N, Potter AS, Paulus MP, Prouty D, Zucker RA, Sher KJ. Demographic, physical and mental health assessments in the adolescent brain and cognitive development study: Rationale and description. Dev Cogn Neurosci. 2018 Aug;32:55-66. doi: 10.1016/j.dcn.2017.10.010.<br>3. Garavan H, Bartsch H, Conway K, Decastro A, Goldstein RZ, Heeringa S, Jernigan T, Potter A, Thompson W, Zahs D. Recruiting the ABCD sample: Design considerations and procedures. Dev Cogn Neurosci. 2018 Aug; 32:16-22. doi: 10.1016/j.dcn.2018.04.004.<br>4. Jernigan TL, Brown SA, Dowling GJ. The Adolescent Brain Cognitive Development Study. J Res Adolesc. 2018 Mar;28(1):154-156. doi: 10.1111/jora.12374.
BiB	1. Born in Bradford. (2023, June 01). https://borninbradford.nhs.uk/<br>2. Born in Bradford Data Dictionary. (2023, June 01). https://borninbradford.github.io/datadict/bib/<br>3. Bird PK, McEachan RRC, Mon-Williams M, Small N, West J, Whincup P, Wright J, Andrews E, Barber SE, Hill LJB, Lennon L, Mason D, Shire KA, Waiblinger D, Waterman AH, Lawlor DA, Pickett KE. Growing up in Bradford: protocol for the age 7-11 follow up of the Born in Bradford birth cohort.<br>4. McEachan, R. R. C., Santorelli, G., Watmuff, A., Mason, D., Barber, S. E., Bingham, D. D., Bird, P. K., Lennon, L., Lewer, D., Mon-Williams, M., Shire, K. A., Waiblinger, D., West, J., Yang, T. C., Lawlor, D. A., Pickett, K. E., & Wright, J. (2024). Cohort Profile Update: Born in Bradford. *International journal of epidemiology*, *53*(2), dyae037. https://doi.org/10.1093/ije/dyae037<br>5. Raynor P; Born in Bradford Collaborative Group. Born in Bradford, a cohort study of babies born in Bradford, and their parents: protocol for the recruitment phase. BMC Public Health. 2008 Sep 23;8:327. doi: 10.1186/1471-2458-8-327.<br>6. Shire K, Andrews E, Barber S et al. Starting School: a large-scale start of school assessment within the 'Born in Bradford' longitudinal cohort [version 1; peer review: 1 approved, 1 approved with reservations]. Wellcome Open Res 2020, 5:47 (https://doi.org/10.12688/wellcomeopenres.15610.1)<br>7. Wright J, Small N, Raynor P, Tuffnell D, Bhopal R, Cameron N, Fairley L, Lawlor DA, Parslow R, Petherick ES, Pickett KE, Waiblinger D, West J; Born in Bradford Scientific Collaborators Group. Cohort Profile: the Born in Bradford multi-ethnic family cohort study. Int J Epidemiol. 2013 Aug;42(4):978-91. doi: 10.1093/ije/dys112.
BIGCS	1. BIGCs. (2023, June 09). http://www.bigcs.com.cn/en_index.html<br>2. The Born in Guangzhou Cohort Study (BIGCS). (2023, June 09). https://clinicaltrials.gov/study/NCT02526901?tab=table<br>3. Qiu X, Lu JH, He JR, Lam KH, Shen SY, Guo Y, Kuang YS, Yuan MY, Qiu L, Chen NN, Lu MS, Li WD, Xing YF, Zhou FJ, Bartington S, Cheng KK, Xia HM. The Born in Guangzhou Cohort Study (BIGCS). Eur J Epidemiol. 2017 Apr;32(4):337-346. doi: 10.1007/s10654-017-0239-x.
CPCD	1. Zhao, L., Shek, D. T. L., Zou, K., Lei, Y., & Jia, P. (2022). Cohort Profile: Chengdu Positive Child Development (CPCD) survey. International journal of epidemiology, 51(3), e95–e107. https://doi.org/10.1093/ije/dyab237
ELFE	1. French Longitudinal Study of Children. (2023, May 31). https://www.elfe-france.fr/en/<br>2. Charles MA, Leridon H, Dargent P, Geay B, the Elfe team. Tracking the lives of 20,000 children: Launch of the Elfe child cohort study. Population & Societies. 2021 Feb;475(2).<br>3. Charles MA, Thierry X, Lanoe JL, Bois C, Dufourg MN, Popa R, Cheminat M, Zaros C, Geay B. Cohort Profile: The French national cohort of children (ELFE): birth to 5 years. Int J Epidemiol. 2020 Apr 1;49(2):368-369j. doi: 10.1093/ije/dyz227.<br>4. Vandentorren S, Bois C, Pirus C, Sarter H, Salines G, Leridon H; Elfe team. Rationales, design and recruitment for the Elfe longitudinal study. BMC Pediatr. 2009 Sep 21;9:58. doi: 10.1186/1471-2431-9-58.
FIN-HIT	1. The Finnish Health in Teens study – Fin-HIT. (2023, May 25). https://www.finhit.fi/<br>2. Figueiredo RAO, Simola-Ström S, Rounge TB, Viljakainen H, Eriksson JG, Roos E, Weiderpass E. Cohort Profile: The Finnish Health in Teens (Fin-HIT) study: a population-based study. Int J Epidemiol. 2019 Feb 1;48(1):23-24h. doi: 10.1093/ije/dyy189.
GenV	1. GenV. (2023, June 09). https://www.genv.org.au/<br>2. Wake M, Goldfeld S, Davidson A. Embedding Life Course Interventions in Longitudinal Cohort Studies: Australia's GenV Opportunity. Pediatrics. 2022 May 1;149(Suppl 5):e2021053509R. doi: 10.1542/peds.2021-053509R.<br>3. Wake, M., Hu, Y.J., Warren, H. et al. Integrating trials into a whole-population cohort of children and parents: statement of intent (trials) for the Generation Victoria (GenV) cohort. BMC Med Res Methodol 20, 238 (2020). https://doi.org/10.1186/s12874-020-01111-x.
Hokkaido Birth Cohort	1. Kishi R, Ikeda-Araki A, Miyashita C, Itoh S, Kobayashi S, Ait Bamai Y, Yamazaki K, Tamura N, Minatoya M, Ketema RM, Poudel K, Miura R, Masuda H, Itoh M, Yamaguchi T, Fukunaga H, Ito K, Goudarzi H; members of The Hokkaido Study on Environment and Children's Health. Hokkaido birth cohort study on environment and children's health: cohort profile 2021. Environ Health Prev Med. 2021 May 22;26(1):59. doi: 10.1186/s12199-021-00980-y.<br>2. Kishi R, Araki A, Minatoya M, Hanaoka T, Miyashita C, Itoh S, Kobayashi S, Ait Bamai Y, Yamazaki K, Miura R, Tamura N, Ito K, Goudarzi H; members of The Hokkaido Study on Environment and Children's Health. The Hokkaido Birth Cohort Study on Environment and Children's Health: cohort profile-updated 2017. Environ Health Prev Med. 2017 May 18;22(1):46. doi: 10.1186/s12199-017-0654-3.<br>3. Kishi R, Kobayashi S, Ikeno T, Araki A, Miyashita C, Itoh S, Sasaki S, Okada E, Kobayashi S, Kashino I, Itoh K, Nakajima S; Members of the Hokkaido Study on Environment and Children's Health. Ten years of progress in the Hokkaido birth cohort study on environment and children's health: cohort profile--updated 2013. Environ Health Prev Med. 2013 Nov;18(6):429-50. doi: 10.1007/s12199-013-0357-3.<br>4. Kishi R, Sasaki S, Yoshioka E, Yuasa M, Sata F, Saijo Y, Kurahashi N, Tamaki J, Endo T, Sengoku K, Nonomura K, Minakami H; Hokkaido Study on Environment and Children's Health. Cohort profile: the Hokkaido study on environment and children's health in Japan. Int J Epidemiol. 2011 Jun;40(3):611-8. doi: 10.1093/ije/dyq071.<br>5. Poudel K, Kobayashi S, Miyashita C, Yamaguchi T, Tamura N, Ikeda-Araki A, Ait Bamai Y, Itoh S, Yamazaki K, Masuda H, et al. Hypertensive Disorders during Pregnancy and Anthropometric Measurement of Children up to 7 Years of Age: The Hokkaido Birth Cohort Study in Japan. International Journal of Environmental Research and Public Health. 2021; 18(20):10951. https://doi.org/10.3390/ijerph182010951
JECS	1. JECS Japan Environment and Children's Study. (2023, June 02). https://www.env.go.jp/chemi/ceh/en/<br>2. Ishitsuka, K., Nakayama, S.F., Kishi, R. et al. Japan Environment and Children's Study: backgrounds, activities, and future directions in global perspectives. Environ Health Prev Med 22, 61 (2017). https://doi.org/10.1186/s12199-017-0667-y.<br>3. Kawamoto, T., Nitta, H., Murata, K. et al. Rationale and study design of the Japan environment and children's study (JECS). BMC Public Health 14, 25 (2014). https://doi.org/10.1186/1471-2458-14-25.

	4.	Michikawa T, Nitta H, Nakayama SF, Yamazaki S, Isobe T, Tamura K, Suda E, Ono M, Yonemoto J, Iwai-Shimada M, Kobayashi Y, Suzuki G, Kawamoto T; Japan Environment and Children's Study Group. Baseline Profile of Participants in the Japan Environment and Children's Study (JECS). J Epidemiol. 2018 Feb 5;28(2):99-104. doi: 10.2188/jea.JE20170018.
	5.	Sekiyama M, Yamazaki S, Michikawa T, Nakayama SF, Nitta H, Taniguchi Y, Suda E, Isobe T, Kobayashi Y, Iwai-Shimada M, Ono M, Tamura K, Yonemoto J, Kawamoto T, Kamijima M; Japan Environment and Children's Study Group. Study Design and Participants' Profile in the Sub-Cohort Study in the Japan Environment and Children's Study (JECS). J Epidemiol. 2022 May 5;32(5):228-236. doi: 10.2188/jea.JE20200448.
Ko-Chens	1.	Jeong KS, Kim S, Kim WJ, Kim HC, Bae J, Hong YC, Ha M, Ahn K, Lee JY, Kim Y, Ha E; Ko-CHENS Study group. Cohort profile: Beyond birth cohort study - The Korean CHildren's ENvironmental health Study (Ko-CHENS). Environ Res. 2019 May;172:358-366. doi: 10.1016/j.envres.2018.12.009.
	2.	Lee E, Baik D, Park Y, Ki M. The current status of health data on Korean children and adolescents. Epidemiol Health. 2017 Dec 26;39:e2017059. doi: 10.4178/epih.e2017059.
Lifelines	1.	Lifelines. (2023, July 11). https://www.lifelines-biobank.com/
	2.	Lifelines catalog. (2023, July 11). https://data-catalogue.lifelines.nl/@molgenis-experimental/molgenis-app-lifelines-webshop/dist/index.html#/shop/
	3.	Scholtens S, Smidt N, Swertz MA, Bakker SJ, Dotinga A, Vonk JM, van Dijk F, van Zon SK, Wijmenga C, Wolffenbuttel BH, Stolk RP. Cohort Profile: LifeLines, a three-generation cohort study and biobank. Int J Epidemiol. 2015 Aug;44(4):1172-80. doi: 10.1093/ije/dyu229.
	4.	Sijtsma A, Rienks J, van der Harst P, Navis G, Rosmalen JGM, Dotinga A. Cohort Profile Update: Lifelines, a three-generation cohort study and biobank. Int J Epidemiol. 2022 Oct 13;51(5):e295-e302. doi: 10.1093/ije/dyab257.
PERSIAN Birth Cohort	1.	PERSIAN Birth Cohort. (2023, July 06). https://persian-bc.family/research#data-collection
	2.	Eghtesad S, Mohammadi Z, Shayanrad A, Faramarzi E, Joukar F, Hamzeh B, Farjam M, Zare Sakhvidi MJ, Miri-Monjar M, Moosazadeh M, Hakimi H, Rahimi Kazerooni S, Cheraghian B, Ahmadi A, Nejatizadeh A, Mohebbi I, Pourfarzi F, Roozafzai F, Motamed-Gorji N, Montazeri SA, Masoudi S, Amin-Esmaeili M, Danaie N, Mirhafez SR, Hashemi H, Poustchi H, Malekzadeh R. The PERSIAN Cohort: Providing the Evidence Needed for Healthcare Reform. Arch Iran Med. 2017 Nov 1;20(11):691-695.
	3.	Poustchi H, Eghtesad S, Kamangar F, Etemadi A, Keshtkar AA, Hekmatdoost A, Mohammadi Z, Mahmoudi Z, Shayanrad A, Roozafzai F, Sheikh M, Jalaeikhoo A, Somi MH, Mansour-Ghanaei F, Najafi F, Bahramali E, Mehrparvar A, Ansari-Moghaddam A, Enayati AA, Esmaeili Nadimi A, Rezaianzadeh A, Saki N, Alipour F, Kelishadi R, Rahimi-Movaghar A, Aminisani N, Boffetta P, Malekzadeh R. Prospective Epidemiological Research Studies in Iran (the PERSIAN Cohort Study): Rationale, Objectives, and Design. Am J Epidemiol. 2018 Apr 1;187(4):647-655. doi: 10.1093/aje/kwx314.
	4.	Zare Sakhvidi, M. J., Danaei, N., Dadvand, P., Mehrparvar, A. H., Heidari-Beni, M., Nouripour, S., Nikukar, H., Daniali, S. S., Saffarieh, E., Noorishadkam, M., Amin, M. M., Mirmohammadkhani, M., Lotfi, M. H., Vaez, A., Mirmohammadi, S. J., Zarean, E., Mojibian, M., Hashemipour, M., Yaghini, O., Rezai, M. S., Esmaeili, A., Fahimzad, A., Hakimi, H., Navaeifar, M. R., Ebrahimi, H. O., Poustchi, H., Malekzadeh, R., & Kelishadi, R. (2021). The Prospective Epidemiological Research Studies in IrAN (PERSIAN) Birth Cohort protocol: rationale, design and methodology, Longitudinal and Life Course Studies, 12(2), 241-262. Retrieved Jul 6, 2023, from https://doi.org/10.1332/175795920X16062247639874
SCHEDULE-P	1.	Shanghai Children's Health, Education and Lifestyle Evaluation, Preschool (the SCHEDULE-P Study). (2023, June 23). https://classic.clinicaltrials.gov/ct2/show/NCT04037761
	2.	Wang X, Zhang Y, Zhao J, Shan W, Zhang Z, Wang G, Jiang Y, Zhu W, Zhang D, He Y, Mao H, Qu J, Zhu Q, Jiang F. Cohort Profile: The Shanghai Children's Health, Education and Lifestyle Evaluation, Preschool (SCHEDULE-P) study. Int J Epidemiol. 2021 May 17;50(2):391-399. doi: 10.1093/ije/dyaa279.
TMM BirThree	1.	Birth and Three-Generation Cohort Study (TMM BirThree Cohort Study). (2023, July 21). https://www.megabank.tohoku.ac.jp/english/research/cohortbiobank/birthree/
	2.	Fuse N, Sakurai-Yageta M, Katsuoka F, Danjoh I, Shimizu R, Tamiya G, Nagami F, Kawame H, Higuchi S, Kinoshita K, Kure S, Yamamoto M. Establishment of Integrated Biobank for Precision Medicine and Personalized Healthcare: The Tohoku Medical Megabank Project. JMA J. 2019 Sep 4;2(2):113-122. doi: 10.31662/jmaj.2019-0014.
	3.	Kobayashi T, Kobayashi M, Minegishi N, Kikuya M, Obara T, Ishikuro M, Yamanaka C, Onuma T, Murakami K, Ueno F, Noda A, Uruno A, Sugawara J, Suzuki K, Kodama EN, Hamanaka Y, Tsuchiya N, Kogure M, Nakaya N, Taira M, Sakurai-Yageta M, Tamahara T, Kawashima J, Goto M, Otsuki A, Shimizu R, Ogishima S, Hashizume H, Nagami F, Nakamura T, Hozawa A, Kobayashi T, Fuse N, Kuriyama S, Kure S, Yamamoto M. Design and Progress of Child Health Assessments at Community Support Centers in the Birth and Three-Generation Cohort Study of the Tohoku Medical Megabank Project. Tohoku J Exp Med. 2023 Jan 20;259(2):93-105. doi: 10.1620/tjem.2022.J103.
	4.	Kuriyama S, Metoki H, Kikuya M, Obara T, Ishikuro M, Yamanaka C, Nagai M, Matsubara H, Kobayashi T, Sugawara J, Tamiya G, Hozawa A, Nakaya N, Tsuchiya N, Nakamura T, Narita A, Kogure M, Hirata T, Tsuji I, Nagami F, Fuse N, Arai T, Kawaguchi Y, Higuchi S, Sakaida M, Suzuki Y, Osumi N, Nakayama K, Ito K, Egawa S, Chida K, Kodama E, Kiyomoto H, Ishii T, Tsuboi A, Tomita H, Taki Y, Kawame H, Suzuki K, Ishii N, Ogishima S, Mizuno S, Takai-Igarashi T, Minegishi N, Yasuda J, Igarashi K, Shimizu R, Nagasaki M, Tanabe O, Koshiba S, Hashizume H, Motohashi H, Tominaga T, Ito S, Tanno K, Sakata K, Shimizu A, Hitomi J, Sasaki M, Kinoshita K, Tanaka H, Kobayashi T; Tohoku Medical Megabank Project Study Group; Kure S, Yaegashi N, Yamamoto M. Cohort Profile: Tohoku Medical Megabank Project Birth and Three-Generation Cohort Study (TMM BirThree Cohort Study): rationale, progress and perspective. Int J Epidemiol. 2020 Feb 1;49(1):18-19m. doi: 10.1093/ije/dyz169.
	5.	Kuriyama S, Yaegashi N, Nagami F, Arai T, Kawaguchi Y, Osumi N, Sakaida M, Suzuki Y, Nakayama K, Hashizume H, Tamiya G, Kawame H, Suzuki K, Hozawa A, Nakaya N, Kikuya M, Metoki H, Tsuji I, Fuse N, Kiyomoto H, Sugawara J, Tsuboi A, Egawa S, Ito K, Chida K, Ishii T, Tomita H, Taki Y, Minegishi N, Ishii N, Yasuda J, Igarashi K, Shimizu R, Nagasaki M, Koshiba S, Kinoshita K, Ogishima S, Takai-Igarashi T, Tominaga T, Tanabe O, Ohuchi N, Shimosegawa T, Kure S, Tanaka H, Ito S, Hitomi J, Tanno K, Nakamura M, Ogasawara K, Kobayashi S, Sakata K, Satoh M, Shimizu A, Sasaki M, Endo R, Sobue K, Tohoku Medical Megabank Project Study Group T, Yamamoto M. The Tohoku Medical Megabank Project: Design and Mission. J Epidemiol. 2016 Sep 5;26(9):493-511. doi: 10.2188/jea.JE20150268.
Young-HUNT4	1.	Questionnaires from the HUNT studies. (2023, July 25). https://www.ntnu.edu/hunt/data/que
	2.	HUNT Databank. (2023, July 25). https://hunt-db.medisin.ntnu.no/hunt-db/variablelist
	3.	Åsvold BO, Langhammer A, Rehn TA, Kjelvik G, Grøntvedt TV, Sørgjerd EP, Fenstad JS, Heggland J, Holmen O, Stuifbergen MC, Vikjord SAA, Brumpton BM, Skjellegrind HK, Thingstad P, Sund ER, Selbæk G, Mork PJ, Rangul V, Hveem K, Næss M, Krokstad S. Cohort Profile Update: The HUNT Study, Norway. Int J Epidemiol. 2023 Feb 8;52(1):e80-e91. doi: 10.1093/ije/dyac095.
	4.	Holmen TL, Bratberg G, Krokstad S, Langhammer A, Hveem K, Midthjell K, Heggland J, Holmen J. Cohort profile of the Young-HUNT Study, Norway: a population-based study of adolescents. Int J Epidemiol. 2014 Apr;43(2):536-44. doi: 10.1093/ije/dys232.

5. Krokstad S, Langhammer A, Hveem K, Holmen TL, Midthjell K, Stene TR, Bratberg G, Heggland J, Holmen J. Cohort Profile: the HUNT Study, Norway. Int J Epidemiol. 2013 Aug;42(4):968-77. doi: 10.1093/ije/dys095.
6. Krokstad S, Sund ER, Kvaløy K, Rangul V, Næss M. HUNT for better public health. Scand J Public Health. 2022 Nov;50(7):968-971. doi: 10.1177/14034948221102309.
7. Rangul, V., Holmen, T. L., Langhammer, A., Ingul, J. M., Pape, K., Fenstad, J. S., & Kvaløy, K. (2024). Cohort Profile Update: The Young-HUNT Study, Norway. *International journal of epidemiology*, *53*(1), dyae013. https://doi.org/10.1093/ije/dyae013
